# Facts for the People

**Published:** 1848-01

**Authors:** H. Crane


### FACTS FOR THE PEOPLE.

BY H. CRANE, D. D. S.

"Doctor, I have come all the way from Lock Lick to consult you respecting my children's teeth."

"Madam, I shall esteem it a privilege to give you any information that I may possess upon that subject."

"I feel greatly obliged for your courteous offer. My children have needed the services of a Dentist for more than a year, but I have delayed for reasons which perhaps it would be useless to name, inasmuch as I am now beyond the reach of their influence."

"Madam, I am prepared to hear those reasons, and as they may have some bearing upon the subject matter of this interview, you will excuse the curiosity that prompts me in requesting that they be frankly given."

"Well, Doctor, I will state them as briefly as possible, but I trust that you will not lay weakness or want of judgment to my charge on account of the influence they exerted over me. About a year or eighteen months since, a man, calling himself a Dentist, called at my house in the famous village of Lock Lick, carrying on his arm a rusty looking market basket, and enquired whether any member of my family needed their teeth plugged, filed, scraped or extracted. I knew that my children needed the services of a dentist, and therefore decided to put the little knowledge that I obtained in early life, while at sundry times I was having operations performed on my own teeth, into immediate requisition. I asked this "Knight of the Turn-key" what was the best material used in plugging teeth—how long each plug would last—what length of time it would require to complete the operation—and what would be the probable expense? He stated that cement was decidedly the best material now known for filling teeth, and that it was introduced in less than half the time that was required to perform the same operation with silver or gold, and that the operation produced no pain, because no excavation was necessary —and with regard to its durability he added, "that depends upon how long the individual lives." He remarked "that the expense was just double that of any other material. He charged fifty cents. This was a little more than many charged, but his experience in the business was so great, as he could easily satisfy me from numerous letters in his possession from Ministers, Judges, Lawyers, Magistrates and Constables, that he was entitled to some little extra consideration.'

"Here he took breath for a moment, and then proceeded.

"'Silver was deemed next best for this purpose, but the difficulty that many experience (though he had experienced none) in making it adhere to the bone, had to considerable extent, excluded it from general use; and the same reason can be urged against the use of gold. They are neither of them as expensive, only costing twenty-five cents a plug, but they will not stick to the bone.'

"I then asked his opinion of the best method of cleansing teeth.

"'O!' he remarks, 'that is a very simple but a beautiful operation. You have only to take a little cotton and, after dipping it into acid, brush it over the surface of the tooth and you have it in an instant as white as pearl. This was the mode adopted by some of the oldest and most respectable dentists, and consequently must be correct.'

"I was afraid to trust my temper longer, so great was my disgust and indignation.

"I told him, in a manner that he immediately comprehended, that his services were not desired in my family. I presume he thought himself very poorly paid for his elaborate desertation on the teeth. He however very soon recovered his self-complacency, and concluded his visit by asking "whether I had any furniture that needed cleansing or varnishing." Now Doctor will you pardon these lengthy reasons for delaying this important matter, and proceed to examine my children's teeth, beginning with Caroline?"

"I will proceed with the examination, Madam, but before I close I shall briefly notice the hero of your interview. I should judge Caroline to be about six years old."

"Within two months of six, Doctor."

"She has all her temporary teeth, which you are aware number ten on the upper and ten on the lower jaw. All teeth that make their appearance after this will never be renewed. They are the last teeth."

"I was not before aware of the exact number of the temporary teeth. But are not those large teeth of George's to be renewed?"

"George is older, and we will attend to him soon Caroline's front under teeth are loose, which is an indication of the speedy progress of her two permanent teeth; but I should let them, however loose, remain until the roots are sufficiently absorbed to be taken out with the fingers or a thread, in order that the process or bone that surrounds them, may not be absorbed by premature extraction, and thereby leave the permanent teeth without the support designed for them. Should the permanent teeth make their appearance, either behind or in front of the temporary teeth, extraction of the temporary teeth is the only alternative to prevent permanent irregularity in the permanent teeth. I discover that something is necessary to be done to these two upper teeth. They are already much decayed, and matter is oozing from under the gum around them. The permanent teeth have scarcely emerged from the pulp or soft state as yet, and the inflammation caused by these two teeth, as indicated by a diseased state of the gums, will injure the surface of the permanent teeth if permitted to remain."

"But Doctor, do you not discover that Caroline's teeth are already decayed, and she has had them scarcely two years yet?"

"Undoubtedly I do, and it is quite common at the present day. I seldom see a child of her age who has not more or less temporary teeth decayed."

"Now, Doctor, you have quite excited my curiosity. I desire to know what you mean by those four short words, "at the present day," on which you laid such particular emphasis? I have often remarked that teeth suffer more "at the present day" from some cause than they did when I was young. My father and mother retained most of their teeth during life. My teeth, I acknowledge, were more frail, but I never heard of such a thing as children's teeth decaying when I was a girl."

"You have touched now upon a subject that requires more time than I have at present, to do justice to its great importance."

"Doctor, I cannot permit you to evade the question in this manner. I have come all the way from Lock Lick for information, and I am determined to save my childrens' teeth if knowledge and skill can do it; for I would not have Caroline, or Frank, or Bell, or Francisco have such a disgusting looking mouth as Janet Vincent has, for every dollar that I have or expect to have. Why, Doctor, it is impossible for me to remain even for a moment in her presence, without heartily wishing that my nose had been placed on the back side of my head. Perhaps I am doing injustice to the girl though, for this is a misfortune for which she is not in fault, therefore I would just add that in every other respect she is a most intelligent and agreeable companion. But if I was a gentleman, Doctor, John Jacob Astor's thirty millions could not gild over this misfortune."

TO BE CONTINUED.

